# Effectiveness of the Sforzesco brace according to the SRS and SOSORT criteria for bracing studies

**DOI:** 10.1186/1748-7161-8-S2-O59

**Published:** 2013-09-18

**Authors:** Monia Lusini, Sabrina Donzelli, Salvatore Minnella, Fabio Zaina, Stefano Negrini

**Affiliations:** 1 ISICO Italian Scientific Spine Institute, Milan, Italy

## Background

Bracing treatment proved to be a useful conservative care treatment for adolescent idiopathic scoliosis (AIS) patients according to a Cochrane review. Prospective observational trials following the Scoliosis Research Society (SRS) criteria for bracing studies and the SOSORT management criteria, can give other evidence on the effectiveness of bracing in AIS. The Sforzesco brace has been used in worse curves, and its efficacy compared to casting

## Purpose

The goal of this study was to check the effectiveness of the Sforzesco brace in AIS treatment according to the SRS and SOSORT criteria. 

## Methods

Design: Prospective cohort nested in a clinical database started in 2003. Population: on July 31, 2010, in our database there were 7,917 patients with scoliosis. Eighty-nine patients (72 females, 17 males) had a Sforzesco brace prescription at first visit and respected the SRS criteria (AIS; age 10 years or older; Risser test 0-2; Cobb degrees 25°-40°; no prior treatment; females less than one year post-menarche). End-of-treatment was defined as medical prescription, or reaching European Risser 3 (corresponding to American Risser 4). Six patients were excluded because they had not yet finished treatment. At start: 12.56±1.25 years of age; 34.16°±4.25° Cobb; ATR 11.11°±3.10°; Trace 7 (IC95 4;10). In all, 81% reached the 2-year follow-up. Treatment: 68 patients were prescribed the brace 23/24 hours/day, 2 22/24, 16 21/24, 3 18/24. All prescriptions included physiotherapic specific exercises (SEAS). All patients were treated until the end of growth. Failures: efficacy analysis (EA): surgery, end of treatment >45°; intent-to-treat (ITT): as EA + drop-outs. 

## Results

Six patients dropped out; 38 patients (45.7%) improved >5° Cobb; 38 (45.7%) did not change; 7 (8.4%) worsened; 4 (4.8%) finished >45°; 2 (2.4%) were fused. EA: 5 failures (6.0%); ITT: 11 failures (12.4%). Patients joining treatment achieved a 4.18°±8.37° Cobb improvement (minimum-31°, maximum 38°), an ATR reduction of 3.28±3.46, and a TRACE improvement of 3 points (IC95 -5; 2.44). 

## Conclusions and discussion

Since this brace is preferred in worst cases, the population was shifted toward high limits of SRS criteria (84.3% >30° at start); nevertheless, this study confirmed the effectiveness of the Sforzesco brace in the treatment of AIS. 
